# Peripheral immune system in aging and Alzheimer’s disease

**DOI:** 10.1186/s13024-018-0284-2

**Published:** 2018-10-03

**Authors:** Wei Cao, Hui Zheng

**Affiliations:** Department of Molecular and Human Genetics, Baylor College of Medicine, Huffington Center on Aging, Houston, TX 77030 USA

**Keywords:** Alzheimer’s disease, Aging, Senescence, Peripheral immune response, Adaptive immune response, Humoral immune response, T cells, T regulatory cells, Immunosignature

## Abstract

Alzheimer’s disease (AD) represents an urgent public health mandate. AD is no longer considered a neural-centric disease; rather, a plethora of recent studies strongly implicate a critical role played by neuroinflammation in the pathogeneses of AD and other neurodegenerative conditions. A close functional connection between the immune system and central nervous system is increasingly recognized. In late-onset AD, aging represents the most significant risk factor. Here, from an immunological perspective, we summarize the prominent molecular and cellular changes in the periphery of aging individuals and AD patients. Moreover, we review the knowledge gained in the past several years that implicate specific arms of the peripheral immune system and other types of immune responses in modulating AD progression. Taken together, these findings collectively emphasize a dynamic role of a concert of brain-extrinsic, peripheral signals in the aging and degenerative processes in the CNS. We believe that a systematic view synthesizing the vast amounts of existing results will help guide the development of next-generation therapeutics and inform future directions of AD investigation.

## Background

Alzheimer’s disease (AD), the most common form of age-associated dementia and neurodegenerative disorder, is a major public health predicament and represents a fundamental scientific challenge. Traditionally, AD has been viewed as a brain-specific disease. Pathologically, AD brains harbor amyloid plaques that contain extracellularly deposited amyloid β (Aβ) from cleaved amyloid precursor protein, and neurofibrillary tangles formed by intracellular accumulation of hyperphosphorylated and misfolded tau protein. These characteristic entities inspired a leading theory that centers on the loss of proteostasis within the brain, which instigates the pathogenic course of AD. The Amyloid Cascade Hypothesis [1–3] has guided numerous studies in the past two decades, which helped reveal insights of the neuronal properties and pathological events initiated by Aβ and subsequently by tau aggregation. However, it is clear that late-onset Alzheimer’s disease (LOAD) is collectively modified by numerous genetic factors that govern diverse cellular and molecular pathways, including many genes involved in the immune responses [4–6]. Consequently, The Neuroinflammation Hypothesis emphasizes the dysregulation of central nervous system (CNS) immune response as a key factor in the etiology of neurodegenerative diseases. In recent years, neuroinflammation is increasingly recognized as an integral and critical contributor in AD pathogenesis [7–9].

The role played by the immune system in AD pathogenesis is prominent but is by no means limited to the brain. Copious evidence from clinical and experimental research suggests an influential, yet largely underappreciated, force in AD pathogenesis: systemic immune signals originating outside the brain. This review will primarily focus on the changes and involvement of the peripheral immune system in aging and AD. Readers interested in the functions of microglia, brain-resident macrophage-like immune cells at the center of neuroinflammation, in aging and AD can refer to other recent publications [10–15]. Here we summarize the current understanding of the profound effects of aging on the systemic components of the host. We further review how different elements of the peripheral immune system and diverse responses modulate the functions of the brain, influence neuroinflammation, and participate in AD pathogenesis with a focus on Aβ-associated events, and discuss implications in the therapeutic development for AD. By uniting multiple influential processes and bridging cross-disciplinary studies, we present a unique systematic perspective to broaden the comprehension on the molecular and cellular players involved in AD pathogenesis.

### Systemic changes associated with aging

#### Inflammaging

**Fig. 1:**
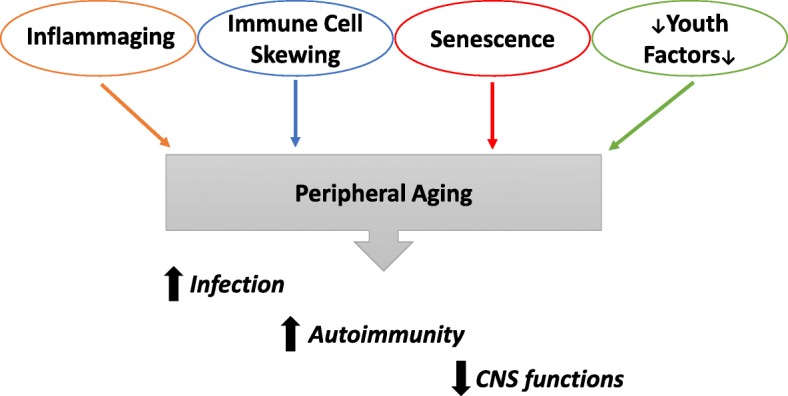
Multifactorial systemic changes are associated with chronological aging and linked to diminished health status. Peripheral aging is promoted by inflammaging, immune cell skewing, senescence, and depletion of youth protective factors. The broad outcome of aging includes increased incidences of infection, autoimmune diseases, and decline of memory and cognitive functions.

As the most significant risk factor for AD, aging itself exerts profound impacts on the immune system and the peripheral tissues (Fig. 1). Innate immunity protects the host by detecting pathogen-associated pattern molecules and activating signaling pathways that result in rapid secretion of cytokines and chemokines, the key soluble immune effector molecules. It has been shown that aging alters the components of innate immunity ranging from the expression of signaling molecules to the behavior of neutrophils, monocytes, dendritic cells, NK cells, etc. [16]. The phenomenon of inflammaging is characterized by chronic low-grade inflammation and functional decline in an aging host [17, 18]. Inflammasomes are intracellular multicomponent structures that enable the secretion of IL-1β and IL-18, important proinflammatory cytokines [19, 20]. The expression of specific inflammasome gene modules in older individuals is associated with higher incidence of elevated blood pressure, arterial stiffness, metabolic dysfunction, oxidative stress and shortened life span [21]. Accordingly, age-related increase in circulating IL-18 is significantly reduced in mice lacking Nlrp3 inflammasome or inflammasome adaptor Asc [22]. Other cytokines and proinflammatory factors have also been associated with age-related decline in physical and cognitive function [23, 24].

#### Immune cell skewing

Aging is associated with increased cell-to-cell transcriptional variability in different tissues, including cells of the hematopoietic lineage [25–28]. Accordingly, aged peripheral immune cells contain highly heterogeneous epigenetic modifications between individuals and from cell to cell, as revealed by single-cell chromatin modification profiling [28]. In addition, substantial alteration in the populations of cells mediating adaptive immunity is well-documented: naïve T cells and circulating B cells are reduced while terminally differentiated T cells are increased in aged individuals [16, 29]. The age-dependent loss of T cell homeostasis has been linked to thymus involution, which is mediated by Nlrp3 inflammasome [22]. Aged naïve T cells possess a less diverse T cell receptor (TCR) repertoire and may switch to a dysfunctional “virtual memory” cell phenotype, which is accompanied by profound epigenetic changes [28–34]. Furthermore, both aged CD4^+^ and CD8^+^ T cells display features of cellular senescence (see below). In aging, B cell receptor repertoire diversity and switched memory B cells are significantly reduced, which is accompanied by a diminished antibody response to antigen challenge. On the other hand, a distinct subset of mature B cells, termed age-associated B cells, accumulates in aged mice and older humans, which may promote inflammation and autoimmunity while inhibiting B cell lymphopoiesis [35, 36]. Beyond blood, aging shifts the functional balance between self-renewal and differentiation in hematopoietic stem cells in the bone marrow and favors the generation of myeloid lineage cells [37]. Overall, an aged immune system renders the host susceptible to infections and increases the incidences of autoimmune and other diseases, which is accompanied by the decline of cognitive functions (Fig. 1).

#### Senescence

During aging, senescent cells accumulate in the body by entering a long-lasting form of cell-cycle arrest triggered by various molecular stressors. Breakthrough studies in recent years have revealed a complex mechanism underlying the biogenesis of these cells and their roles in diverse physiological and pathological conditions (reviewed in [38–40]). The involvement of senescent cells in host immunity is closely linked to their acquired ability to secrete proinflammatory cytokines, a phenomenon termed the senescence-associated secretory phenotype (SASP) [18]. SASP is induced primarily by NF-κB in response to DNA damage, oncogenic stress and developmental cues, which initiates the transcription of a host of genes including IL-6, IL-1β, TNF-α, IL-8, etc. [41, 42]. Given the proinflammatory nature of SASP, cellular senescence in multiple organs and tissues significantly promotes inflammaging in the elderly [18, 43]. As mentioned earlier, naïve T cell senescence is associated with a loss of immune diversity. Concomitantly, terminally differentiated senescent T cells expand and over-express cytotoxins and proinflammatory cytokines, contributing to the hyperinflammatory status of the host. Several groups recently demonstrated a remarkable functional restoration in multiple tissues and organs of the mice after selective ablation of senescent cells by genetic manipulation or treatment with senolytic compounds [44]. The classical senescent cells have not been widely identified in aging CNS beyond the description of SASP+ astrocytes [45]. The impact of senescent cell ablation on brain function is yet to be demonstrated.

#### Diminished youth factors

Aging not only induces the generation of harmful products, it also depletes the molecules that keep the host young [46]. Shared circulation between young and old mice by way of parabiosis has demonstrated the ability of old blood to transfer aging phenotypes to peripheral tissues and the brain of young mice, and vice versa [47]. Immune factors enriched in aged plasma, such as CCL11 and B2M, have been shown to negatively affect neurogenesis and memory [47, 48]. In contrast, growth and differentiation factor 11 (GDF-11) supplementation to aged mice was shown to benefit the heart and muscle and increase neurogenesis [49–51]. However, the expression and function of GDF-11 remains controversial and further studies are needed to confirm its role in host rejuvenation [52, 53]. Nevertheless, a proof-of-concept study demonstrated a near complete restoration of synaptic and neuronal proteins in aged amyloid precursor protein (APP)-expressing mice after exposure to circulating young blood or receiving plasma transfusion [54]. Improved working and associative memory occurred after young plasma administration in the absence of changes in amyloid load [54]. A renewed search for systemic brain rejuvenating factors led to the recent identification of tissue inhibitor of metalloproteases 2 (TIMP2), which is present at highest levels in the human umbilical cord blood, over young and old adult blood [55]. As reported, TIMP2 not only promotes neuronal plasticity inside the brain likely through modulating extracellular matrix, but also affects hippocampal synaptic plasticity from systemic circulation. Although direct translation of the findings to human diseases is uncertain at this point, these studies illustrate a dynamic, complex, and powerful crosstalk between the periphery and the brain.

### Molecular alterations in the circulation of AD patients

Early observational studies and meta-analyses detected an association between elevated levels of inflammatory factors and increased risk for AD development [56, 57]. In the past decade, many proteomic surveys have been performed mostly on human plasma and occasionally on serum samples, using diverse technical platforms, ranging from filter-based arrayed sandwich ELISA, 2D-gel electrophoresis coupled with liquid chromatography and mass spectrometry, Luminex xMAP, Myriad RBM, to SOMAscan multiplexed protein technology (reviewed in [51, 58]). Although the list of top altered proteins overlapped and differed between individual studies, a consensus pathway analysis revealed significant changes of multiple immunological processes, among which Complement and Coagulation Cascades and Cytokine-Cytokine Receptor Interaction are ominously affected [58]. Noteworthy is that both are centrally involved in the neuroinflammation inside the brains of AD patients and AD animal models [59, 60].

Although over 20% of people diagnosed with mild cognitive impairment (MCI) develop dementia over the subsequent 3 to 10 years, it remains difficult to predict the disease course for individual patients. By examining the plasma communicome, *i.e.* a group of signaling proteins involved in intracellular communication, *Ray et al* reported an initial success in identifying MCI patients who are *en route* to develop AD based on a panel of 18 affected proteins consisting primarily of chemokines and growth factors [61]. Subsequent studies using more advanced detection technologies and analyzing a larger cohort of patients established a subset of 10-13 plasma proteins, including several apolipoproteins, chemokines and growth factors, whose abundance is associated with disease severity and progression of AD [62, 63]. More recently, a simple combination of two plasma markers (Apo A-II and cortisol) and four cerebrospinal fluid (CSF) proteins (fibroblast growth factor 4, calcitonin, heart-type fatty acid binding protein, and TRAIL-R3) was shown to be valuable in predicting midterm progression from MCI to AD dementia [64]. Hence, molecular changes in the blood may be useful to probe the ongoing neurodegeneration in the brain. Before any of these markers are adopted as blood biomarkers for clinical prognosis, their robustness, accuracy, and reproducibility need to be firmly established.

### Involvement of peripheral immune cells in brain homeostasis and aging

Like other organs, the CNS is under regular immune surveillance. While microglia cells reside inside the parenchyma, small but significant numbers of T cells, B cells, NK cells, and dendritic cells migrate into and populate the meninges and choroid plexus (CP) that form a direct interface with the brain parenchyma [65]. A series of studies have established an indispensable role played by CD4^+^ T cells in maintaining the cognitive and behavioral capacity of naive mice [66–68]. In particular, CD4^+^ T helper cells and their secreted cytokines after undergoing polarization facilitate memory function and social behaviors. Both IFNγ-producing (T_H_1) and IL-4-producing (T_H_2) CD4^+^ T cells reside in the meninges under steady-state conditions. IFNγ supports neuronal circuits that are important for social behavior [69] and IL-4 facilitates learning by regulating meningeal dendritic cells and stimulating BDNF expression in astrocytes [70]. Therefore, T cells and their secreted cytokines are critically involved in preserving the homeostatic functions of the brain (Table 1).

**Table 1:** T cell subsets maintain homeostatic brain functions

Subset/Cytokine	Location	Effects on brain function	Specificity	Mechanism	Reference
T_H_1/IFN_Ɣ_	Meninges	↑ Social behavior	bulk	IFN_Ɣ_ signaling activation in inhibitory neurons increases GABAergic currents	[69]
T_H_2/IL-4	Meninges	↑ Learning and memory	bulk	Restricts meningeal myeloid cell activation and promotes neutrophic factor expression	[70]
↑T_H_2:T_H_1 ratio	CP of Aged Mice	↓ Cognitive capacity	CNS antigens	IL-4 induces while IFN_Ɣ_ inhibits CCL11 expression by epithelia cells	[73]

CP is an epithelial tissue located within the ventricles of the brain [71, 72]. As the site of the blood-CSF barrier, CP plays indispensable roles in maintaining brain homeostasis by secreting neurotropic factors into CSF, clearing toxic molecules including Aβ from CSF, and facilitating immune surveillance and leukocyte trafficking [71, 72]. In naïve mice, more than 50% of CD4^+^ and CD8^+^ T cells in the brain reside in the stroma of the CP. Most of these T cells have an effector memory phenotype, including T_H_1, T_H_2 and Tregs, and recognize CNS antigens [73]. However in aged CP, a distorted T_H_1: T_H_2 balance arises, which results in increased chemokine CCL11 expression, decreased permeability to leukocytes owing to the differential effects of IL-4 and IFNɣ on CP epithelial cells, and compromised cognition [73–75] (Table 1). Type I interferon (IFN-I) is a major innate immune cytokine commonly involved in host defense as well as autoimmune conditions. Interestingly, aged CP of both human and mouse display strong IFN-I signaling, which was shown to counter the function of IFNγ (i.e. type II interferon), disrupt the infiltration of monocytes, and hamper cognitive functions and hippocampal neurogenesis [76]. Hence, aging disrupts the balance of brain-homing peripheral immune cells, which negatively impacts essential CNS functions.

### Involvement of peripheral immune cells in AD pathogenesis

#### Innate immune cells

Inside the brain, ongoing AD pathology leads to the differentiation of microglia into a novel type associated with neurodegenerative diseases with altered molecular expression profile and limited phagocytic capacity [77, 78]. The precise origin of plaque-surrounding amoeboid myeloid cells has long been debated, owing to the technical challenge of distinguishing infiltrating myeloid cells from locally-activated microglia. Nevertheless, recent evidence suggests that peripherally-derived macrophages can engraft the brain and maintain a unique functional and transcriptional identity in CNS [79]. Infiltrating peripheral myeloid cells have been demonstrated to participate in Aβ clearance, leading to the notion that monocytes are superior phagocytes when traversed inside the AD brain [80, 81]. However, such a conclusion is challenged by studies showing that infiltrating peripheral myeloid cells, in replacing ablated microglia, adopt a microglia-like phenotype in the brain, *i.e.* with limited phagocytic capacity, likely influenced by local molecular cues [82, 83]. Whether additional signals are needed to boost their phagocytic function *in situ* requires further investigation.

Rare variants in Triggering Receptor Expressed on Myeloid cells 2 (TREM2) increase the risk of developing AD by 2~3 fold. Significant TREM2-dependent phenotypes in mouse models of AD shed light on the role of TREM2 in regulating neuroinflammation during AD pathogenesis [84]. Besides CNS upregulation, expression of TREM2 mRNA and protein are increased in the peripheral leukocytes of AD patients and correlated with cognitive deficits and hippocampal atrophy [85–88]. It is unclear if elevated peripheral TREM2 expression has functional impact on disease pathogenesis or merely reflects the ongoing systemic inflammation of AD.

Neutrophils are the most abundant myeloid cells in human peripheral blood and participate critically in protective innate immunity. Neutrophils have been detected in the brain parenchyma of 5xFAD and 3xTg-AD mice and were shown to promote amyloid plaques and tau tangles as well as cognitive decline [89]. Yet, a separate study showed that long term treatment of 3xTg-AD mice with a TNF-α modulator led to increased neutrophil infiltration in the brain, which coincided with improved learning and memory, and reduced tau and amyloid pathology [90]. Thus, the functional features of infiltrating neutrophils remain to be fully elucidated.

#### Adaptive immune cells

Distinct from innate immunity, adaptive immune responses protect the host in a manner that is long-lasting and antigen-specific. Although innate immune responses at the site of brain inflammation are well-established, the role played by adaptive immunity in AD remains vague, primarily due to the scarcity of T cells or B cells inside the parenchyma. Nevertheless, a recent large-scale genome-wide association study strongly implicated the involvement of both adaptive and innate immunity in AD [91]. In addition, the association of a single-nucleotide polymorphism (SNP) of MHC class II HLA-DRB5 with AD implies a potential T cell-mediated process [92]. To assess the overall function of the adaptive immunity, two groups analyzed severely immunocompromised transgenic mice expressing human mutant APP and obtained conflicting results. PSAPP:Rag2^-/-^ mice lacking B and T cells showed reduced Aβ pathology accompanied by highly phagocytic microglia [93], whereas 5xFAD:Rag2^-/-^:Il2rg^-/-^ mice lacking B, T and NK cells displayed exaggerated Aβ plaque deposition and increased neuroinflammation [94]. While the two mouse models do differ in several aspects, the primary reason for the discrepant observations is unclear. Given the memory and behavior impairment in Rag2^-/-^ mice [68, 95, 96], how this may confound Aβ-associated functional decline was not addressed.

#### B cells in adaptive immunity

Humoral immune response (*i.e.* antibody production) by B cells to amyloid β has been studied for over 20 years. It was first discovered that immortalized B cells from the peripheral blood of an AD patient secreted antibodies that specifically recognize Aβ peptide [97]. Circulating anti-Aβ antibody at various levels has since been detected in the blood of human subjects with or without AD [98–101]. The groundbreaking study by Schenk et al, in which Aβ immunization prevented the development of amyloid plaques, neuritic dystrophy and gliosis in PDAPP mice, demonstrated the therapeutic potential of B cell-mediated immune response [102]. However, human vaccination trial AN1792 based on the same principle had to be halted due to life-threatening encephalitis, which was later attributed largely to the pathogenic autoimmune T_H_1 response stimulated by active immunization (see next section). Subsequent studies in animal models demonstrated the ability of individual Aβ-specific antibody clones to attenuate AD pathogenesis without the involvement of T cells, lending support for the clinical trials to passively immunize AD patients with anti-Aβ antibodies. However, such a strategy has yielded mixed outcomes with several trials still ongoing (reviewed by [103–105]; also see section *Passive Immunotherapy*)).

Besides the humoral response specific to proteins associated with AD pathology, the peripheral immunoglobulin repertoire is dysregulated in AD. Spontaneously secreted by B cells without exogenous stimulation, natural antibodies are abundant in normal sera, typically poly- or self-reactive, and fulfill important functions of targeting pathogens and removing cellular and molecular waste in the body [106]. The levels of natural IgG recognizing self-antigens was shown to be influenced by age, sex and disease [107]. Significant reduction of the number of autoantibodies was detected in the sera from patients with AD, Parkinson’s disease (PD), and multiple sclerosis, a neuroinflammatory condition [107]. The cause and functional significance of such a decrease is unclear. Unexpectedly, a recent time-course global proteomic analysis of APP^NL-F^ mice reported an inverse correlation between Aβ burden in the brain and IgM levels in the blood [108]. Again, the implication of this finding remains to be seen. Nevertheless using a panel of autoantibodies as biomarkers, DeMarshall et al. were able to differentiate mild-moderate AD patients from age-matched controls, MCI, and other neurological disorders with high confidence [109].

Another insight on the role of immunoglobulin in AD came from the study by Marsh et al. [94]. Increased mouse IgGs were detected in association with microglia in the brains of 5xFAD mice. Lacking specificity for Aβ, these proteins were shown to engage with microglia Fc receptor, induce an activating signaling pathway, and stimulate phagocytosis of Aβ, which lead to a decreased plaque load. Although the antigen-specificity of the infiltrating IgG was not studied in this study, the protective effect of mouse IgG is reminiscent of the beneficial influence previously reported with intravenous Immunoglobulin (IVIg), a pooled human IgG product [110–112]. Whether any specific subset of the natural IgGs confers enhanced neuroprotection remains to be investigated.

#### T cells in adaptive immunity

Inside the post-mortem brains of AD patients, both CD4^+^ and CD8^+^ T cells were detected, occasionally next to the neuritic plaques or microglia [113–115]. Similarly, increased numbers of T cells have been found infiltrating the brain parenchyma of multiple transgenic mice expressing mutant human APP [116, 117]. In one study, significant fractions of infiltrating T cells were found to produce IFNγ and IL-17 in APP/PS1 brain [117]. However, Ferretti et al. reported that infiltrating T cells consistently display an inactivated phenotype with reduced IFNγ production and lack of local proliferation and do not co-localize with amyloid plaques in three AD models (ArcAβ, APP/PS1 and Tg2576) [116]. In line with this, peripheral CD4^+^ T cells were found hyporesponsive to Aβ peptides in Tg2576 mice, which resulted in a defective Aβ-specific antibody response [118]. In humans, an early study reported unresponsiveness to Aβ40 peptide by peripheral lymphocytes from AD patients [119]. However, peripheral T cells reactive to Aβ42 peptide, which is more immunogenic than Aβ40, was later found significantly increased in AD patients and older adults over middle-aged healthy controls [120]. T regulatory cells (Tregs) are a crucial T cell subset that suppresses effector immune responses and maintains immune tolerance. AD and Down syndrome patients both display Aβ plaque accumulation and similarly have increased Aβ-specific IL-10-producing Treg cells in their blood [121]. Elevated Treg levels and suppressive activities in the periphery of AD patients were reported by other groups [122–124], which is affirmed by the increased FoxP3^+^ Tregs in the spleens of 5xFAD mice [125].

Given the distinct and powerful roles played by T helper subsets in numerous diseases, it would be vital to dissect how different T_H_ cell populations specifically modulate AD. In the APP/PS1 model, adoptive transfer of Aβ-specific T_H_1 cells, but not T_H_2 or T_H_17 cells, led to their homing to the brain and worsened AD pathology along with impaired cognitive function and exaggerated microglia activation [117]. The pathogenic and plaque clearing roles of T_H_1 cells were demonstrated in an earlier study, in which, post-Aβ vaccination, Aβ-specific IFNγ-producing T cells infiltrated the brain of J20 mice to clear amyloid plaques but induced meningoencephalitis, mirroring the pathogenic events of the failed human vaccination trial AN1792 [126]. However, direct cerebrospinal injection of Aβ-specific T_H_1 cells led to amyloid plaque clearance and increased neurogenesis in the absence of autoimmunity, implying an added impact of peripheral T_H_1 cells in APP/PS1 model [127]. On the other hand, adoptively transferred Aβ-specific T_H_2 cells, while with no evidence of brain filtration, improved working memory of APP/PS1 mice in conjunction with reduced systemic inflammation and vascular amyloidosis [128]. Therefore, Aβ-specific T_H_1 and T_H_2 effector subsets seemingly alter the Aβ pathology in distinct ways (Table 2).

**Table 2:** Aβ-specific T cell subsets regulate AD pathogenesis in experimental models

Subset	Location	AD Model	Specificity	Effects on pathogenesis	Reference
T_H_1	Parenchyma	APP/PS1	Aβ	Adoptively Transferred Cells Increased Microglia Activation And Aβ deposition	[117]
T_H_1	Parenchyma	J20 with Aβ peptide vaccination	Aβ	Migrated to Aβ plaques with increased clearance, while inducing transient meningoencephalitis	[126]
T_H_1	Parenchyma	APP/PS1	Aβ	Cells injected to cerebrospinal fluid ventricle migrated to Aβ plaques, increased Aβ clearance and promote neurogenesis	[126, 127]
T_H_2	Outside the brain	APP/PS1	Aβ	Adoptively transferred cells improved working memory, decreased microgliosis and reduced plasma cytokines. No effect on plaque load inside the parenchyma but reduced vascular amyloidosis	[128]

Substantial yet contradictory impacts of Tregs on AD pathogenesis have been demonstrated by recent studies in several experimental models (Table 3). By way of antibody-mediated depletion, adoptive transfer of purified cells, and low-dose IL-2-induced peripheral expansion, multiple groups independently provided experimental evidence that collectively suggested an overall protective role of Tregs in restoring memory deficits, reducing plaque load and decreasing microglia activation as well as inflammation in several APP transgenic models [129–132]. These results are consistent with the impaired learning and memory in IL-2^-/-^ mice, which lack functional Tregs [133]. Whether Tregs convey neuroprotection in an antigen-specific manner is not clear at this time.

**Table 3.** Treg cells regulate AD pathogenesis in experimental models

Subset	Location	AD model	Specificity	Effects on pathogenesis	Reference
T_reg_	Systemic	APP/PS1	bulk	Transient depletion of Treg accelerated cognitive decline; increased Treg with low-dose IL-2 treatment restored cognitive functions	[129]
T_reg_	Systemic	3xTg	bulk	Adoptively transferred cells improved cognitive functions and reduced Aβ deposition; long-term Treg depletion resulted in exacerbated spatial learning deficits, Aβ plaque load and microgliosis	[130]
T_reg_	Systemic and Parenchyma	App/PS1 ΔE9	bulk	AAV-IL-2 expression within the brain induced Treg expansion and astrocyte activation, reduced Aβ plaque and improved synaptic plasticity and spine density	[131]
T_reg_	Systemic	AβPPswe/PS1 Δ E9	bulk	Adoptively transferred cells improved cognitive function, while reducing Aβ deposition, microgliosis and systematic inflammation	[132]
T_reg_	Systemic	5xFAD APP/PS1	bulk	Transient depletion or pharmacological inhibition of Treg lead to Aβ plaque clearance, neuroinflammation and reversal of cognitive decline. It affected CP with increased recruitment of peripheral monocytes and Tregs to Aβ plaque	[125]
T_reg_	Systemic	5xFAD	bulk	Anti-PD1 treatment stimulated IFN_Ɣ_-dependent systematic immune response, which resulted in the recruitment of peripheral monocytes and Tregs to Aβ plaque, clearance of plaque, and improvement of cognitive performance. Repeated treatments maintained a long-lasting beneficial effects	[134]
T_reg_		ThyAPP/PS1_m146L_ ThyAPP/PS1_A246E PD-APP_	bulk	Anti-PD1 treatments had no effect on amyloid pathology nor induced infiltration of peripheral monocyte into the brain	[135]

On the other hand, two consecutive reports from Michal Schwartz’s group strongly argued for detrimental effects of Tregs in AD pathogenesis [125, 134]. They first demonstrated that 5xFAD mice have reduced expression in choroid plexus of molecules critical for transepithelial migration, which correlated with the loss of IFNγ signaling, a condition similar to brain aging [125]. This led to a hypothesis that a dysregulated CP exacerbates AD pathogenesis by blocking protective immune cell infiltration. Since Tregs are the primary suppressor of IFNγ-producing T_H_1 cells, depleting or disabling Treg function was shown to restore IFNγ signaling in CP, increase the number of peripheral immune cells in the parenchyma, clear amyloid plaques, and rescue memory and behavior defects in 5xFAD mice [125, 134]. In more detail, Baruch et al. perturbed the Treg population by various experimental schemes, which included selective depletion in 5xFAD:FoxP3-DTR mice with diphtheria toxin, chemical treatments to boost or diminish the functionality of Tregs or stimulate their induction, and administrating antibody against PD1 to break Treg suppression in 5xFAD and/or APP/PS1 models (Table 3).

At this time, it is unclear whether these directly opposing results reported on Tregs are affected by the genetic background of the AD models or other factors. Nevertheless, a T cell-based intervention with anti-PD1 antibody, which is largely analogous to the cancer checkpoint immunotherapy, was proposed as a translatable approach of AD therapeutics based on the findings by Baruch et al. [135]. However most recently, a joint study by three pharmaceutical companies questioned the effectiveness of systemic PD-1 blockade in modifying amyloid burden in several AD mouse models, where the authors also failed to detect monocyte infiltration into the brain ([136]; Table 3). Hence, the mechanism of how Tregs modulate Aβ pathology remains to be fully elucidated and the pre-clinical support for PD-1 blockade approach in AD patients is weak at this time.

### Peripheral immune responses modify AD pathogenesis

#### Pathogens

The brain can readily respond to inflammatory signals originated from the periphery. For example, peripheral administration of microbial mimetics, such as LPS and polyI:C, that induce strong systemic inflammatory response, exacerbates neurodegeneration [137, 138]. Microglia are shown to be highly receptive to these treatments and the resulting enhanced neuroinflammation compels the disease pathogenesis [139]. Living in a bustling world, humans are exposed to exogenous infectious agents, cohabit with a complex microbiome, and are afflicted increasingly by chronic ailments as we age. Immune system is the frontline protection against microbial infections. When the infectious agents are not well controlled, HIV patients develop dementia that is highly analogous to AD, West Nile Virus infection leads to long term cognitive impairment, and periodontitis exacerbates the cognitive decline in AD patients, etc. [140–142].

#### Microbiota

Mammals host trillions of microbes that reside in the barrier tissues such as gut, skin, and lung. Research in recent years have revealed a dynamic interaction between the microbiome and the immune system and the pathogenic outcomes of dysbiosis [143, 144]. Microbes in the gut profoundly affect the development of microglia [145] and effectively prime microglia in the brain via secreted short-chain fatty acids [146], highlighting an eminent gut-brain axis [147]. Several studies have suggested that gut microbiota plays a vital role in the pathogenesis of PD via modulating neuroinflammation [148, 149]. To understand its potential relevance to AD, two groups surveyed the gut microbe taxa in elderly individuals. AD patients have reduced microbial diversity and harbor a distinct bacteria genera, which is correlated with changes in CSF biomarkers [150]. A similar association was made between inflammatory bacteria and proinflammatory circulating cytokines in patients with cognitive impairment [151]. Consistent with this, a shift in the gut microbiota was detected in APP/PS1 mice compared with non-transgenic controls. Depletion of microbiota by antibiotic treatments or germ-free host re-derivation resulted in reduced Aβ plaque load in conjunction with altered microglia morphology [152, 153]. Intriguingly, transient perturbation of microbial diversity in APP/PS1 mice shortly after birth can result in long-lasting alteration of brain Aβ pathology [154]. Although microbiota can modulate CNS profiles in mice [155], whether and how gut microbiota affect cognition and memory in AD models has yet to be fully revealed. This area of research is being actively pursued and will surely help illuminate how such key environmental factor modulate AD risk and progression.

#### Chronic inflammation

A recent prospective survey of 1,633 participants revealed a positive association between midlife peripheral inflammation and shrinkage of brain volume in late-life [156]. Not unexpectedly, a genetic epidemiology study identified SNPs that are shared between LOAD and common chronic inflammatory diseases, such as psoriasis, type 1 diabetes and Crohn disease [92]. Chronic conditions with inflammatory traits, such as vascular diseases and diabetes, are well-known comorbidities of AD. Aging is also associated with higher incidents of autoinflammatory diseases, such as rheumatoid arthritis (RA) and psoriasis, two diseases also co-morbid with AD [157, 158]. RA patients receiving anti-inflammatory treatments have overall reduced risk of developing AD, and peripheral neutralization of TNF-α, a key cytokine driving RA pathogenesis, was shown effective in AD animal models [159]. Therefore, diverse systemic inflammatory responses play a crucial role in modulating AD pathogenesis.

### Therapeutic applications in AD by harnessing the immune system

Deemed the greatest global challenge for health and social care in the 21^st^ century, dementia affected an estimated 47 million people globally in 2015, a number which may increase to 131 million by 2050 as the world’s population ages. The task to develop effective treatments for AD is exceedingly daunting, due to the multifaceted nature of the disease, lack of sensitive and definitive biomarkers, incomplete understanding of the pathogenic and regulatory pathways, dearth of disease stage-specific molecular targets, and technical difficulties for effective CNS drug delivery. Nevertheless, a range of strategies harnessing our knowledge of immune responses and aging/AD pathogenesis are being actively pursued.

#### Passive immunotherapy

Despite the strong implication of Aβ peptide in AD, strategies targeting monomeric Aβ failed in late stage AD populations [104, 160]. Along the protein misfolding pathway, soluble oligomeric assemblies are recognized as the primary pathogenic species of misfolded proteins [161, 162]. Although antibodies against different forms of aggregated Aβ are detected in the peripheral blood of healthy individuals, the levels of these antibodies decrease with normal aging and decline further with progression from mild to severe AD [163, 164]. Inoculation of antibodies recognizing aggregated Aβ effectively modified amyloid plaque pathology and rescued cognitive decline in AD animal models [165–167]. Given the protective humoral response in the host, a “reverse translational medicine” approach was devised. A fully humanized IgG1 mAb reactive to aggregated but not monomeric Aβ, aducanumab, was obtained from a screen of healthy advanced-age donors with normal cognition who presumably harbor naturally developed protective mAbs against Aβ. Preliminary results from a double-blind, placebo-controlled phase 1b trial with aducanumab showed reduced brain Aβ plaques in a dose- and time-dependent manner and a trend of slowing clinical decline in AD patients [167]. Two large-scale phase 3 trials with aducanumab are ongoing and will be completed in 2022. In addition to Aβ, antibodies specific to tau protein are also being actively evaluated for their clinical potentials [160].

One significant impediment for the success of passive immunotherapy for AD is the insufficient entry of antibodies into the CNS. It was estimated that only approximately 0.1% of peripherally-administered antibody can enter the brain in the presence of an intact blood brain barrier (BBB), a network of vascular endothelial cells, pericytes and astrocytes, which significantly blunted the drug availability inside the brain [168, 169]. Different strategies are being devised to improve CNS delivery of therapeutic antibodies. A bispecific antibody design with one arm recognizing transferrin receptor, a surface molecule highly expressed by BBB endothelial cells, greatly enhanced transcytosis of the antibody across the BBB and increased the antibody levels inside the brain [170, 171]. On the other hand, transient opening of the BBB can be achieved by focused ultrasound treatment, which was shown to result in the reduction of amyloid burden in AD models and enhanced the protective effect of a tau-specific antibody *in vivo* recently [172–175].

#### Targeting immune molecules

Consistent with the notion of a pathogenic influence of inflammation, early epidemiological studies suggested that long term use of anti-inflammatory drugs was associated with reduced risk of developing AD [176, 177]. This led to multiple clinical trials designed to suppress general inflammation, which included using nonsteroidal anti-inflammatory drugs, statins, low dose-prednisone, and NF-κB blocker, etc.; however, no clinical benefit has been demonstrated in AD patients (reviewed in [178, 179]). Intravenous immunoglobulins have been used clinically for treating various autoimmune and infectious diseases and exhibited protective effects in AD animal models, as discussed earlier [110, 111]. Yet, clinical trials with IVIg failed to show positive effect [180–182].

Given the complex role played by immune cells and responses inside and outside the brain, a deeper understanding of the molecular mechanisms is a prerequisite for translational success. Therapies targeting specific immune molecules that are crucial in AD pathogenesis or protection presumably offer improvement over non-specific interventions. TNF-α is a proinflammatory cytokine highly implicated in many peripheral inflammatory diseases as well as AD. Peripheral TNF-α neutralization was effective in reducing Aβ plaques and neuronal dysfunction in an AD model [159]. A small, double-blind, placebo-controlled phase 2 trial with Etanercept, a decoy receptor for TNF-α, reported a tolerated response [183]. Yet, future trial with large patient cohort is necessary to establish the effectiveness of this strategy. On the other hand, administration of the cytokine Granulocyte-Macrophage Colony-Stimulating Factor (GM-CSF) was shown effective in improving memory in aged mice, reducing brain amyloidosis and reversing the cognitive impairment in AD mice, and associated with improved cognition in cancer patients [55, 184, 185]. A small number of AD patients treated with Leukine®, recombinant human GM-CSF, reported rapid improvement of cognitive functions in a double-blind, placebo-controlled phase 2 trial [186].

#### Anti-aging strategies

Besides disease-specific targeting, slowing down the clock of age-related systemic decline should be beneficial for a wide spectrum of diseases, including AD. Although most studies were carried out in rodents, the exciting findings on the power of young blood and molecules with capacity to rejuvenate the old offer a novel prospect of therapeutics [46]. An ongoing phase I Plasma for Alzheimer’s Symptom Amelioration (PLASMA) trial examines the safety, tolerability and feasibility of infused blood plasma from young donors to patients with mild to moderate Alzheimer’s disease [187]. Alternatively, via selective destruction of senescent cells, senolytics have emerged recently as a novel class of anti-aging agents in animal studies. In the coming years, therapeutic potential to target senescence in humans will surely be explored (reviewed in [44]).

## Conclusions

**Fig. 2:**
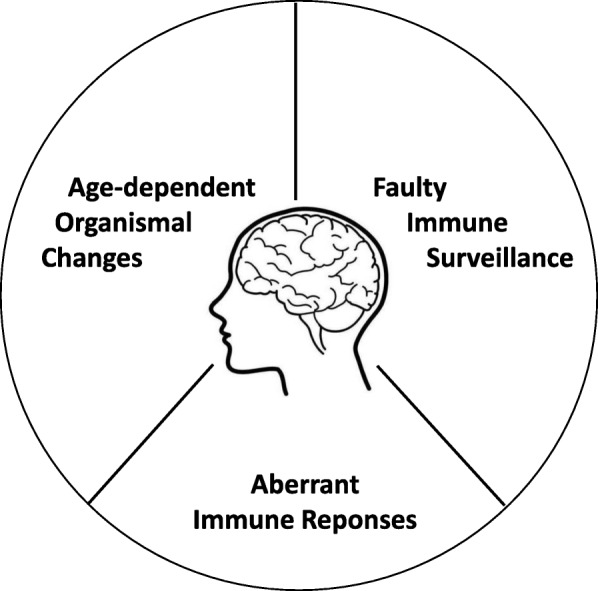
AD pathogenesis is influenced by multiple systemic factors. Aging-induced systemic changes, surveillance by various immune cell populations, and influence of altered systemic immune responses all influence the onset and progression of AD. Understanding the interplay between these elements and AD pathogenesis is crucial for the successful intervention of the disease.

We have summarized the knowledge gained in recent years on the changes of the periphery in aging and Alzheimer’s disease and the emerging significance of the peripheral immune system and their responses in modulating AD pathogenesis. It is thus apparent that the prevailing neuro-immune connection is not limited to localized neuroinflammation inside the brain. Here we emphasize the importance of diverse signals from the periphery to modify the course of AD pathogenesis (Fig. 2). The systemic influences comprise a multitude of age-dependent organismal changes, protective and pathogenic immune cell surveillance, and ongoing immune responses to environmental factors and self-derived molecules. These elements operate jointly and dynamically with the ongoing brain-intrinsic aging and degenerative processes. We believe such understanding helps comprehend the vast amount of existing literature and expand our perspectives in AD research.

Despite the latest immense interest in understanding the role of immune system modulation in AD, some major knowledge gaps remain. Below we identify six key areas that the field should address in the future:

## Contribution of aging to AD

Given the importance of aging in LOAD, it is not surprising that AD pathology *in vivo* is modulated highly by immune responses in an age- and disease-state dependent manner [188, 189]. This poses a significant problem since the development of AD has been mostly studied in adult, but not necessarily advanced-age, animals so far. With the advent of increasingly refined knock-in and transgenic AD animal models [190, 191], analysis of properly aged hosts will offer opportunity to evaluate the physiologically relevant contributing factors in AD pathogenesis and to test the efficacy of targeted regimen for translational potential. In the coming years, it would also be critical to establish and dissect how senescence within and outside CNS partakes in AD pathogenesis.

## A systematic understanding of LOAD

Genetic studies of LOAD have identified numerous immune-related risk factors [4–6]. The involvement of implicated genes and related pathways in AD has been investigated in genetically engineered mouse models in recent years [84, 189, 192–195]. It is important to note that these genes and pathways are expressed at elevated levels both inside the brain and in the periphery in aging and/or AD, such as TREM2, inflammasome, and complement cascade as discussed earlier. Since the in vivo studies focused primarily on events within CNS, the contribution of simultaneously altered peripheral immune compartment is largely ignored. Recently identified as genetic risk factors in the Chinese population, risk variants of both GCH1 and KCNJ15 have altered expression in the peripheral immune cells of AD subjects, suggesting apparent functional effects through the immune system [196]. Examination of the peripheral leukocytes and their alterations together with the cells inside the brain should provide further insights on how the immune system comprehensively influences AD pathogenesis. With the same goal, it would also be necessary to examine the effects of lineage-specific gene knockouts to thoroughly dissect the immune compartments critical for the assigned gene function.

## Adaptive immune system in AD pathogenesis

Despite genetic evidence implicating adaptive immunity in AD [91, 92], it remains ill-defined how adaptive immune cells with limited presence inside the parenchyma exert their effects on AD pathologies and cognitive functions. Nevertheless, T cells have been shown to participate in other neurodegenerative diseases, such as PD and amyotrophic lateral sclerosis [197, 198]. As reviewed earlier, many inconsistent results have been reported on the impacts of T cell subsets on CNS pathogenesis in Aβ-based experimental models. Thus, it is critical in the future to reconcile the main findings and differentiate the neuro-protective molecules, cells, and responses from the pathogenic ones, while elucidating the context-dependent functions influenced by specific host factors. Noticeably missing at this time is the examination of tau-specific T cells and the possible involvement of Treg cells in tau pathology, a major gap in understanding the participation of the adaptive immune arm in AD pathogenesis. Despite the technical challenge to study rare cells, new technologies such as high-dimensional single-cell analysis should significantly improve the quantification and classification of diverse immune cell populations in the AD brain. Whether T and B cells, self-reactive or bystanders, afford protective immune surveillance or pathogenic immune attack requires thorough delineation.

## Peripheral immune responses in modulating AD

Despite strong clinical implications, how peripheral immune responses influence AD progression remains enigmatic. Though useful and informative, current animal models of AD, however, have severe limitations in their capacity to facilitate immunological inquiries. The animals are usually analyzed after life-long housing in the absence of natural microbial pathogens, which may result in reduced host fitness and poor disease resistance [143]. Given the importance of gut microbiota, alternative experimental modeling that incorporates the elements of peripheral conditioning is imperative for dissecting the immune responses in AD. One fundamental question is whether peripheral responses alone are sufficient to initiate the process of neuroinflammation and eventually drive neuropathology under any circumstances. Equally important is to understand whether peripheral events can imprint CNS with long-lasting alterations, a notion highlighted by the detected microglia memory formed upon peripheral LPS administrations [199]. On the other hand, high-dimensional and functional peripheral immune profiling of AD patients may reveal much needed insights into ongoing peripheral responses, which may help stratify the patients for targeted therapeutic interventions.

## Neurovascular interface in AD

The brain is dynamically circulated not only by blood vessels, but also via a perivascular network and meningeal lymphatic vessels [200, 201]. In AD, it yet has to be revealed how peripheral immune cell populations traverse through these neurovascular networks to enter, remain in, and exit the brain, and more importantly, the functional outcome of such events. It is equally unclear how specific components of the neurovascular barrier is differentially regulated by the inflammatory milieu of AD. Understanding the selectivity of gateway trafficking for leukocytes and the associated regulatory mechanism is critical not only for dissecting the crosstalk between the periphery and CNS, but also for devising therapeutic strategies to modify such interactions.

## Neuro-immune feedback mechanism in AD

The immune system and the nervous system share several key features: both regulate physiological homeostasis and protect the host from threats; both are composed of heterogeneous cell populations with extensive functional specialization; and both use synapses and soluble factors for cell-to-cell communication and memory formation [202, 203]. With expression of receptors for cytokines and neural transmitters, immune cells and nerve cells crosstalk and cooperate to maintain the homeostasis of the host. Although we have focused on the effects of peripheral inflammation on CNS, it is known that CNS can exert control of peripheral immune responses through spinal sympathetic and brain stem vagus nerve efferent signaling [204, 205]. For example, the vagus-nerve-based circuit inhibits TNF-α production in the spleen via the splenic nerve, acetylcholine-synthesizing T cells, and response by macrophage to acetylcholine [206]. Among the brain regions that integrate and regulate neuro-immune reflex, both thalamus and hypothalamus harbor pathological changes in AD patients [207, 208]. Therefore, it would be interesting to investigate whether the CNS regulatory circuit is compromised in AD, which may result in dysregulated peripheral immune responses and consequently a feedforward loop for systemic inflammation.

Overall, a new era of discovery compels us to fully grasp the many molecular and cellular players that partake the neuro-immune communications in AD and other neurodegenerative diseases under aging conditions. Better deciphering the influence of the immune system on the brain will surely provide further insights and guidance for the development of next-generation therapeutics.

## Abbreviations

AD: Alzheimer’s disease
APP: Amyloid precursor protein
Aβ: Amyloid β
BBB: Blood brain barrier
CNS: Central nervous system
CSF: Cerebrospinal fluid
GDF-11: Growth and differentiation factor 11
IFN-I: Type I interferon
IVIg: Intravenous Immunoglobulin
LOAD: Late-onset Alzheimer’s disease
SASP: Senescence-associated secretory phenotype
SNP: Single-nucleotide polymorphism
TCR: T cell receptor
Tregs: T regulatory cells
TREM2: Triggering Receptor Expressed on Myeloid cells 2
