# Correction to: Peripheral immune system in aging and Alzheimer’s disease

**DOI:** 10.1186/s13024-018-0290-4

**Published:** 2018-10-24

**Authors:** Wei Cao, Hui Zheng

**Affiliations:** Department of Molecular and Human Genetics, Baylor College of Medicine, Huffington Center on Aging, Houston, TX 77030 USA

## Correction

The original article [1] contained several small omissions and errata contained in Tables 2 and 3; these errors have now been corrected.
